# Risk of recurrence after neoadjuvant chemotherapy and transoral robotic surgery in patients with oropharynx cancer that avoid adjuvant radiation

**DOI:** 10.1002/cam4.7146

**Published:** 2024-04-05

**Authors:** Alisha R. Pershad, Punam G. Thakkar, Joseph F. Goodman, Arjun Joshi, Seth M. Steinberg, Clint T. Allen, Charalampos S. Floudas

**Affiliations:** ^1^ Division of Otolaryngology‐Head and Neck Surgery The George Washington University School of Medicine & Health Sciences Washington DC USA; ^2^ Astrix Technology, LLC, contractor to Biostatistics and Data Management Section, Office of the Clinical Director National Cancer Institute, National Institutes of Health Bethesda Maryland USA; ^3^ Surgical Oncology Program, Center for Cancer Research National Cancer Institute, National Institutes of Health Bethesda Maryland USA; ^4^ Center for Immuno‐Oncology, Center for Cancer Research National Cancer Institute, National Institutes of Health Bethesda Maryland USA

**Keywords:** adjuvant radiotherapy, de‐escalation, neoadjuvant chemotherapy, oropharynx cancer, transoral surgery

## Abstract

**Background:**

De‐escalation strategies for newly‐diagnosed p16‐positive oropharyngeal squamous cell carcinoma (p16+ OPSCC), aim to reduce treatment‐related morbidity without compromising disease control. One strategy is neoadjuvant cisplatin and docetaxel chemotherapy (NAC + S) before transoral robotic surgery, with pathology‐based risk‐adapted adjuvant treatment.

**Methods:**

We examined the recurrence‐free survival (RFS) for patients who received NAC + S.

**Results:**

Comparing outcomes in 103 patients between 2008 and 2023, 92% avoided adjuvant treatment and showed significantly higher 2‐year recurrence‐free survival (RFS) compared to those with adjuvant treatment (95.9% vs. 43.8%, *p* = 0.0049)

**Conclusion:**

Our findings suggest that pathology‐based risk‐adapted omission of adjuvant treatment following NAC + S does not appear to elevate recurrence risk and that NAC may identify patients with favorable tumor biology, yielding a 2‐year RFS probability exceeding 95% without adjuvant treatment. Further, the study identifies a patient subset experiencing disease recurrence despite triple modality therapy. Despite limitations, including a retrospective design and modest sample size, the data advocate for controlled NAC + S studies.

## Graphical abstract

In appropriately selected patients with p16+ oropharyngeal squamous cell cancer, the administration of chemotherapy before surgery may lead to pathologic responses that allow the omission of postoperative adjuvant radiotherapy. In this retrospective study, we find that this omission did not adversely impact the recurrence‐free survival of patients.

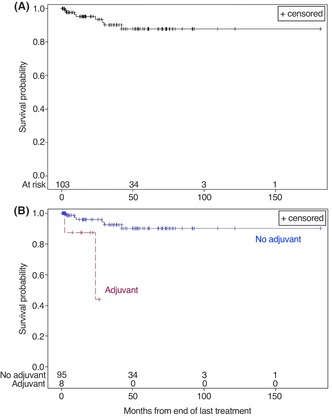

## 1 INTRODUCTION

Treatment of newly‐diagnosed p16‐positive oropharyngeal squamous cell carcinoma (p16+ OPSCC) in accordance with National Comprehensive Cancer Network Guidelines results in excellent recurrence‐free survival.^1^, ^2^ Treatments often include radiation therapy that can result in dysphagia, xerostomia, pain, and other associated morbidity.^3^, ^4^ Treatment de‐escalation aims to reduce long‐term treatment‐associated morbidity while maintaining non‐inferior oncologic control.^2^, ^5^

Neoadjuvant cisplatin and docetaxel chemotherapy before transoral robotic surgery (NAC + S) for newly‐diagnosed p16+ OPSCC is a de‐escalation strategy that allows for complete avoidance of surgical pathology‐based risk‐adapted adjuvant radiation therapy in many patients.^6^, ^7^ Further, 5‐year disease‐free survival (DFS) compared favorably to a historical cohort of similar patients undergoing primary concurrent chemoradiotherapy (CRT).^6^ Yet, concern over whether increased risk of recurrence exists in NAC + S patients who avoid risk‐adapted adjuvant treatment when it might have been indicated without NAC. To address this concern, we performed a retrospective study to compare RFS in patients with newly‐diagnosed p16+ OPSCC treated with NAC + S with or without risk‐adapted adjuvant treatment.

## 2 METHODS

### 2.1 Study population

This study was approved by the George Washington University School of Medicine and Health Sciences Institutional Review Board (NCR234857) and informed consent exemption was granted. Age at diagnosis, sex, race and ethnicity, smoking status, clinical and pathologic TNM and overall stage (AJCC8), treatment modalities received, pathological complete response (pCR) and recurrence during the surveillance period were reviewed for patients with newly‐diagnosed p16+ OPSCC treated with NAC + S ± risk‐adapted adjuvant treatment^6^, ^7^ in a single institution between 2008 and 2023.

### 2.2 Outcomes and statistical analysis

Recurrence Free Survival (RFS) was defined as time between date of last treatment and date of last follow‐up/recurrence. The probability of RFS was estimated using the Kaplan–Meier method. A log‐rank test and a hazard ratio were used to test differences between groups. Pathological complete response probabilities were calculated along with Wilson 95% confidence intervals (CIs). Survival analyses were conducted using SAS, version 9.4 software (SAS Institute Inc).

## 3 RESULTS

**FIGURE 1:**
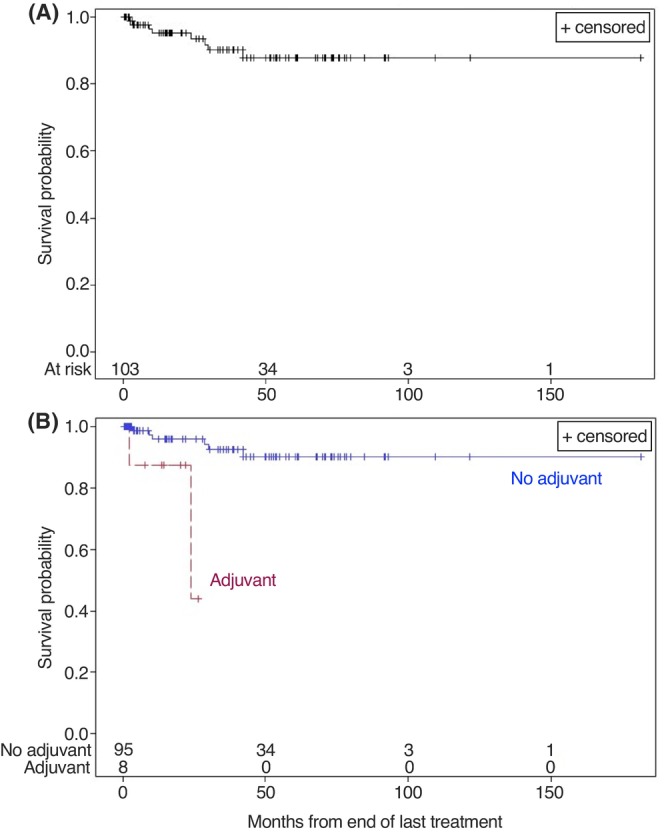
Kaplan–Meier survival curves show recurrence‐free survival (RFS) in months from the end of last treatment for (A) all patients or (B) stratified by delivery of adjuvant treatment.

One hundred and three patients were studied. Mean age was 62.4 years, 87.4% were men. Table 1 lists patient characteristics. Median potential follow‐up time was 44.5 months (interquartile range 19.3–77.6 months). Ninety‐two percent (95/103) avoided any adjuvant treatment. Considering all patients, the percentage with pCR following NAC was 50.5% (95% CI 40.7–60.5%) and had a 2‐year RFS probability of 93.5% (95% CI 84.9–97.3%) and a 5‐year RFS probability of 87.8% (95% CI 76.7–93.9%). In the group of patients that avoided adjuvant treatment, the percentage with pCR was 53.9% (95 CI% 43.6–63.9%). In the group that received adjuvant treatment, the percentage with pCR was 12.5% (95 CI% 6.4–47.0%). Patients that avoided risk‐adapted adjuvant treatment had a much greater 2‐year RFS probability compared to those that received adjuvant treatment (95.9%, 95% CI 87.7–98.7% vs. 43.8%, 95% CI 1.1–86.1%, respectively, *p* = 0.0049, Figure 1; hazard ratio: 8.73 (95% CI: 1.42–53.57)).

**TABLE 1:** Demographic, clinical, and treatment characteristics.

Characteristic	Number	(%) (*n* = 103)
Age at diagnosis, year		
Mean (SD)	62.4	(9.0)
Median (IQR)	62.0	(55.0–68.0)
Range	39.0–83.0	
Sex		
Female	13	12.6
Male	90	87.4
Race		
White	77	74.8
Black/African American	17	16.5
Asian	2	1.9
Other	6	5.8
Unknown	1	1.0
Ethnicity		
Non‐Hispanic/Latino	91	88.3
Hispanic/Latino	3	2.9
Decline to answer	3	2.9
Unknown	6	5.8
Smoking		
Never	63	61.2
Former	35	34.0
Current	5	4.9
Primary site		
Tonsil	58	56.3
Base of tongue	43	41.7
Unknown primary	2	1.9
T (clinical)		
T0	3	2.9
T1	18	17.5
T2	61	59.2
T3	18	17.5
T4	3	2.9
N (clinical)		
N0	6	5.8
N1	96	93.2
N2	1	1.0
AJCC overall stage (eighth Ed.)		
1	81	78.6
2	19	18.4
3	3	2.9
Treatment		
NAC + S	95	92.2
NAC + S + RT/CRT	8	7.8
NAC + S + RT	6	5.8
NAC + S + CRT	2	1.9
T (pathologic)		
T0	73	70.9
T1	23	22.3
T2	5	4.9
T3	1	1.0
TX	1	1.0
N (pathological)		
N0	68	66.0
N1	31	30.1
N2	3	2.9
N2a	1	1.0
Median potential follow‐up, m (IQR)	44.5	(19.3–77.6)
Recurrence		
No	95	92.2
Yes	8	7.8

Abbreviations: AJCC, American Joint Committee on Cancer; CRT, chemoradiotherapy; NAC + S, neoadjuvant chemotherapy with transoral robotic surgery; RT, radiotherapy.

## 4 DISCUSSION

Following curative‐intent surgery for patients with newly‐diagnosed p16+ OPSCC, patients with high‐risk pathologic features receive adjuvant treatment that aims to reduce the risk of recurrence. Here, we observed two‐year RFS probability of >95% in patients that were treated with NAC + S alone, indicating that NAC may select patients with favorable tumor biology that are at low risk of recurrence, even when adjuvant treatment is totally avoided. The observed RFS of patients treated with NAC + S alone is similar to that observed in the low‐ and intermediate‐risk arms of the ECOG 3311 study.^2^ This work also identifies a subset of patients with newly‐diagnosed p16+ OPSCC that develop disease relapse despite triple modality therapy (NAC, surgery and adjuvant radiation). Additional studies are needed to understand the biologic features driving this poor prognosis.

Limitations of our study include the retrospective design, short follow‐up period for some patients, small sample size overall and especially for patients receiving adjuvant treatment. Despite these limitations, this data suggests that prospective, controlled NAC + S clinical studies that allow for risk‐adapted determination of the need for adjuvant treatment are not placing patients at increased risk of recurrence if adjuvant treatment is avoided.

## AUTHOR CONTRIBUTIONS

**Alisha R. Pershad:** Data curation (lead); formal analysis (supporting); writing – original draft (supporting); writing – review and editing (supporting). **Punam G. Thakkar:** Resources (equal). **Joseph Goodman:** Resources (equal). **Arjun Joshi:** Resources (equal). **Seth Steinberg:** Formal analysis (lead); writing – review and editing (supporting). **Clint Allen:** Conceptualization (lead); data curation (supporting); formal analysis (supporting); methodology (supporting); writing – original draft (supporting); writing – review and editing (equal). **Charalampos S. Floudas:** Conceptualization (equal); data curation (supporting); formal analysis (supporting); methodology (equal); writing – original draft (lead); writing – review and editing (lead).

## FUNDING INFORMATION

The authors declare no funding.

## CONFLICT OF INTEREST STATEMENT

None.

## ETHICS STATEMENT

George Washington University School of Medicine and Health Sciences Institutional Review Board (NCR234857).

## Data Availability

The data underlying this article will be shared on reasonable request to the corresponding author.
